# Neuropathic Cranial Pain Phenotypes After Craniotomy: A Large, Single-Center Retrospective Cohort Study

**DOI:** 10.3390/medicina62050840

**Published:** 2026-04-28

**Authors:** Shachar Zion Shemesh, Jose Asprilla, Paz Kelmer, Omri Cohen, Itay Goor-Aryeh, Yotam Hadari, Zvi R. Cohen, Lior Ungar

**Affiliations:** 1 Department of Neurosurgery, Sheba Medical Center, Ramat Gan 5262100, Israel; shemesh.shachar@gmail.com (S.Z.S.);; 2 Gray Faculty of Medicine, Tel Aviv University, Tel Aviv 6139001, Israel; 3 Pain Center, Sheba Medical Center, Ramat Gan 5262100, Israel; 4 Dina Recanati School of Medicine, Reichman University, Herzelia 4610101, Israel

**Keywords:** post-craniotomy headache, neuropathic cranial pain, occipital neuralgia, scar neuroma, chronic postsurgical headache

## Abstract

*Background and Objectives*: Chronic headache after craniotomy is common and may include neuropathic subtypes (scar neuroma pain, occipital neuralgia). However, no large series has quantified these phenotypes. We conducted a single-center retrospective review (*n* = 5624 adult craniotomy patients) to estimate the prevalence of post-craniotomy neuropathic pain and to describe its characteristics. *Materials and Methods*: Institutional records were screened to identify craniotomy patients referred to a multidisciplinary pain clinic (*n* = 272). Eligible cases were reviewed in tiers: (1) exclusion of primary headache and noncranial pain; (2) identification of “probable neuropathic cranial pain” based on documented neuropathic features (lancinating/scalp pain, trigger tenderness, dermatomal distribution); and (3) subgroup categorization into occipital neuralgia-like, supraorbital/supratrochlear neuralgia-like, and scar-site neuropathic pain phenotypes. The supraorbital/supratrochlear subgroup was defined by frontal or frontotemporal postoperative pain in the supraorbital region, local tenderness or Tinel-like hypersensitivity over the supraorbital/supratrochlear course, and/or response to supraorbital–supratrochlear nerve block. Data extracted included demographics, timing (surgery to pain referral), pain characteristics, and treatments (blocks, radiofrequency, medications). *Results*: Of 5624 craniotomy patients, 272 (4.8%) had pain clinic encounters. The initial review identified 124 cases with chronic post-craniotomy headache requiring follow-up; after detailed chart classification, *probable neuropathic cranial pain* was present in 111 cases (2% of the cohort). Among the 111 probable neuropathic cranial pain cases, the dominant regional phenotype was occipital neuralgia-like pain. In addition, eight patients (7.2%) demonstrated a supraorbital/supratrochlear neuralgia-like phenotype, predominantly after frontal or frontotemporal craniotomies. Scar-site neuropathic pain frequently coexisted with both regional phenotypes, supporting a partially overlapping spectrum rather than mutually exclusive categories. The median time from surgery to pain referral was several months (≈12–18 months). Management commonly included occipital nerve blocks (±steroid); some patients received pulsed radiofrequency ablation of the occipital nerves, and most were trialed on neuropathic analgesics (gabapentinoids, SNRIs, etc., according to neuropathic pain guidelines). *Conclusions*: A clinically meaningful subset of post-craniotomy patients develops chronic neuropathic cranial pain, most commonly with occipital, supraorbital/supratrochlear, or scar-related features. Because most postoperative headaches are managed through neurosurgical follow-up and improve without pain clinic referral, the present cohort likely underestimates the true burden of neuropathic post-craniotomy pain while enriching for its most refractory neuralgic presentations. This is nevertheless the subgroup that must be recognized, discussed with patients, studied prospectively, and targeted in future prevention strategies.

## 1. Introduction

Post-craniotomy headache (PCH) is a well-known but under-recognized complication of brain surgery. Although craniotomy is often presumed “painless” relative to other surgeries, acute postoperative head pain is surprisingly common, with some series reporting moderate–severe pain in 60–90% of patients in the first days after craniotomy [1,2,3,4,5,6]. Moreover, a substantial minority of patients develop *chronic* headaches. For example, Kaur et al. (2000) found that 12% of temporal lobectomy patients had headaches persisting one year after surgery [7,8,9,10,11]. Chronic PCH is distressing and affects quality of life, yet it is often misclassified or mismanaged.

Recent reviews have begun to tease apart clinical *phenotypes* of PCH. De Gray [2] described five main patterns, the most notable being *scar-related neuropathic pain* and *occipital neuralgia-like headache*. Scar-related neuropathic pain presents as focal lancinating pain at the surgical incision, possibly due to cutaneous nerve injury or neuroma formation. Occipital neuralgia-like PCH features lancinating pain in the C2 dermatome (posterior scalp), often after suboccipital approaches. Case reports and small series support these concepts: chronic occipital headaches after retrosigmoid acoustic neuroma resection respond to occipital nerve decompression [12,13,14,15,16,17,18,19,20], and scar neuromas have been identified as headache triggers. However, large-scale data on how frequently these neuropathic phenotypes occur is lacking.

A parallel neuropathic mechanism may occur after frontal, frontotemporal, pterional, or anterior skull-base approaches, where injury or entrapment of the supraorbital and supratrochlear nerves may produce a focal frontal-orbital neuralgia-like pain syndrome.

Additionally, patients with PCH often experience long delays before receiving appropriate treatment. For example, Gottschalk et al. emphasized that neurosurgeons frequently underuse opioids, believing that acute pain is minimal [6]. Occipital neuralgia after trauma or surgery is increasingly treated with nerve blocks or neuromodulation; however, post-craniotomy neuropathic headache remains understudied.

Despite growing recognition of persistent post-craniotomy headache as a meaningful source of postoperative morbidity, most prior studies have approached this condition as a broadly defined pain outcome rather than as a set of mechanistically distinct neuropathic syndromes. As a result, clinically relevant phenotypes, such as occipital neuralgia-like pain, scar-site neuropathic pain, and anterior frontal neuralgia-like syndromes, may remain grouped under non-specific postoperative headache labels. This limits both diagnostic precision and treatment selection, particularly in referral populations, in which focal tenderness, dysesthesia, triggerability, or response to targeted nerve blockade may indicate a peripheral neuropathic generator rather than a non-specific chronic headache disorder.

This distinction is also important from a classification perspective. While the International Classification of Headache Disorders, 3rd edition (ICHD-3), provides criteria for conditions such as occipital neuralgia and persistent headache attributed to craniotomy, it does not fully capture the mixed or overlapping neuropathic cranial pain presentations encountered in postoperative neurosurgical practice. Scar-mediated pain and supraorbital/supratrochlear neuralgia-like syndromes after frontal or frontotemporal approaches remain underdescribed in large clinical cohorts. A phenotype-oriented framework may therefore help bridge the gap between existing diagnostic categories and real-world post-craniotomy pain presentations.

We therefore performed a retrospective, single-center cohort study of 5624 adult craniotomy patients to identify and characterize neuropathic cranial pain phenotypes. Using a staged (tiered) classification approach, we explored three recurring phenotypic patterns: occipital neuralgia-like pain, supraorbital/supratrochlear neuralgia-like pain, and scar-site neuropathic pain. We aimed to (a) quantify these phenotypes, (b) describe their pain features and management (nerve blocks, medications), and (c) propose operational definitions and inclusion criteria for future prospective studies. Our analysis focuses on a subset of craniotomy patients who required specialized pain management, thereby enriching for neuropathic cases and reflecting a “real-world” referral population.

## 2. Materials and Methods

### 2.1. Study Design and Cohort

We performed a retrospective chart review of all adult patients (age ≥ 18 years) who underwent open cranial surgery (craniotomy or craniectomy) at our tertiary center between 2010 and 2024. The initial cohort (*n* = 5624) was derived from hospital procedure coding and then de-duplicated and cross-referenced with hospital pain clinic records [10]. Patients seen in our multidisciplinary pain clinic with any postoperative headache-related complaint were flagged for detailed screening (*n* = 272). 

At our institution, most postoperative headache complaints are initially managed through neurosurgical follow-up by the operating surgeon and improve or resolve without referral to a pain specialist. Referral to the pain clinic is typically reserved for persistent, treatment-resistant, or clinically complex pain, particularly when neuropathic or neuralgiform features are suspected. Accordingly, the present cohort was not intended to capture the full spectrum of post-craniotomy headache but rather the more refractory end of the clinical spectrum.

### 2.2. Inclusion/Exclusion Tiers

**Figure 1:**
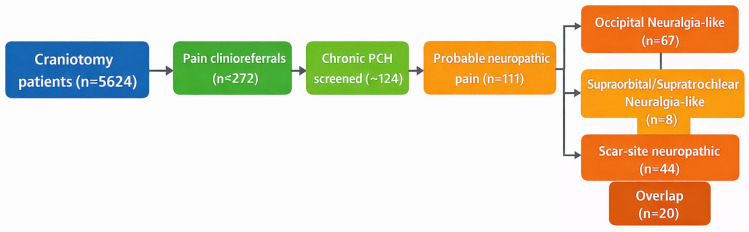
Study flowchart and phenotypic classification of probable post-craniotomy neuropathic cranial pain. Of 5624 patients undergoing craniotomy, 272 underwent postoperative pain clinic evaluation. Following staged chart review, 124 patients were identified as having chronic post-craniotomy headache or cranial pain, and 111 met criteria for probable neuropathic cranial pain. Within this cohort, occipital neuralgia-like pain was the predominant regional phenotype (*n* = 67), whereas a smaller subgroup demonstrated a supraorbital/supratrochlear neuralgia-like phenotype (*n* = 8). Scar-site neuropathic pain (*n* = 44) was treated as an overlapping mechanistic phenotype rather than as a mutually exclusive regional subtype.

We developed a three-tier classification protocol (Figure 1). Tier 1 excluded cases with non-craniotomy causes (e.g., preexisting primary headache disorder without change, scalp or lumbar puncture headache, cancer-related headache, or infection-related headache). Tier 2 identified patients with chronic post-craniotomy headache persisting for > 3 months. Tier 3 applied prespecified chart-based neuropathic criteria. “Probable neuropathic cranial pain” was defined as post-craniotomy headache with at least two of the following features: (i) lancinating, stabbing, or shooting pain within a cranial nerve territory; (ii) focal scalp allodynia, trigger-point tenderness, or scar hypersensitivity; (iii) documented sensory disturbance, such as hyperalgesia, dysesthesia, or pinpoint neuropathic pain; and (iv) temporary improvement after targeted local anesthetic nerve block. This classification framework was informed by ICHD-3 diagnostic concepts for occipital neuralgia and by established neuropathic pain management principles, while recognizing that postoperative cranial pain presentations may not fit neatly into a single existing formal headache category [15]. Two investigators (a neurosurgeon and a pain specialist) independently reviewed all records. Disagreements were resolved by consensus.

### 2.3. Phenotypic Classification

Patients classified as having “probable neuropathic” pain were subtyped as follows:

- **Occipital neuralgia-like:** Unilateral occipital/neck pain radiating toward the vertex or front, often in the C2 dermatome; focal occipital tenderness or Tinel sign; response to GON/LON block [14].
- **Scar-site neuropathic pain:** Pain at or near the craniotomy scar with neuropathic features (burning, lancinating); point tenderness at a scar neuroma; allodynia along the wound edge. Some patients met both categories (overlap).
- **Supraorbital/supratrochlear neuralgia-like:** Postoperative frontal, supraorbital, brow, or peri-orbital pain following frontal, frontotemporal, pterional, or anterior skull-base craniotomy, with neuropathic features such as lancinating or burning quality, focal tenderness or Tinel-like hypersensitivity over the supraorbital/supratrochlear course, sensory disturbance in the frontal scalp, and/or temporary improvement after supraorbital–supratrochlear nerve block.

Because scar-site hypersensitivity frequently coexisted with regional nerve-territory pain, scar-site neuropathic pain was treated as an overlapping mechanistic phenotype rather than as a mutually exclusive regional category.

### 2.4. Data Collection

For each included patient, we extracted demographics (age, sex), craniotomy details (indication, approach), and timelines—the date of surgery and the first pain clinic visit—to calculate lag time. Headache characteristics (distribution, laterality, quality) were recorded from clinical notes. Treatments administered (e.g., GON/LON nerve blocks, supraorbital/supratrochlear nerve blocks, radiofrequency ablation of occipital nerves or scar neuromas, and neuropathic medications) were tabulated. We paid special attention to the use of neuropathic medications (gabapentin, pregabalin, SNRIs, amitriptyline) as an indicator of guideline-based management [15].

Because this was a retrospective chart-based study, phenotypic assignment relied on the quality and consistency of clinical documentation rather than on prospectively applied standardized neuropathic pain instruments. Accordingly, we intentionally used the term “probable neuropathic cranial pain” to reflect a structured but non-prospective classification process. The goal of this framework was not to establish definitive diagnostic criteria but rather to identify clinically coherent postoperative neuropathic phenotypes suitable for descriptive analysis and future prospective validation.

## 3. Results

### 3.1. Cohort and Case Ascertainment

Of 5624 craniotomy patients, *n* = 272 (4.8%) had at least one postoperative headache evaluation in the pain clinic. After applying exclusion criteria (Tier 1), 124 patients had chronic PCH warranting detailed review. Tier 3 classification identified *probable neuropathic cranial pain* in 111 patients (2.0% of the original cohort). Table 1 summarizes the cohort counts.

### 3.2. Demographics and Clinical Features

Of the 111 neuropathic cases, the mean age was 55 years, and 60% were female. The common surgical indications included tumor resection (vestibular schwannomas, meningiomas) and aneurysm clipping. Pain was unilateral in 85% of cases. In the occipital phenotype group, patients reported sharp, stabbing pain in the posterior scalp or neck, radiating to the forehead. All had focal occipital scalp tenderness; 90% had temporary relief with greater or lesser occipital nerve blocks. In the scar phenotype group, pain was localized to the craniotomy or subtemporal incision site, often with painful nodules (suggestive of neuromas). Many described “burning/stabbing” pain along the wound edge. Some had trigger-point hypersensitivity documented.

A smaller but clinically distinct subgroup demonstrated a supraorbital/supratrochlear neuralgia-like phenotype. Using a conservative chart-based definition, 8 of 111 patients (7.2%) met the criteria for this subgroup. These cases occurred predominantly after frontal, frontotemporal, or anterior skull-base craniotomies and were characterized by frontal or brow pain, supraorbital tenderness, Tinel-like hypersensitivity, and partial response to supraorbital/supratrochlear blockade. In several cases, this phenotype coexisted with adjacent scar-site pain or ipsilateral occipital hypersensitivity, underscoring the mixed regional nature of post-craniotomy neuropathic pain.

Occipital cases often had pain triggered by neck movement or pressure on the occiput, consistent with occipital neuralgia criteria [7]. Scar cases frequently reported exacerbation with touch to the scar and had sensory deficits around the scar. Overall, 20 patients met both phenotypic criteria (e.g., pain at the scar extending into the occipital distribution), demonstrating phenotype overlap. This overlap suggests that some patients have complex neuropathic pain involving multiple nerve branches (Figure 1).

### 3.3. Timeline and Management

The median interval from craniotomy to first pain-clinic evaluation was prolonged, on the order of 9–18 months postoperatively, with some patients presenting years after the index surgery. This delay suggests chronicity; in contrast, acute postoperative pain usually resolves or is treated earlier [6]. Once identified, management patterns were broadly consistent with contemporary neuropathic headache practice. Most patients with regional neuropathic phenotypes underwent targeted peripheral nerve blocks. Occipital cases were typically managed with GON/LON blockade, whereas frontal-orbital cases were treated with supraorbital/supratrochlear blockade, sometimes combined with occipital blockade in mixed phenotypes. Some received adjunctive therapies (e.g., botulinum toxin injections) as reported in small series for persistent PCH.

**Figure 2:**
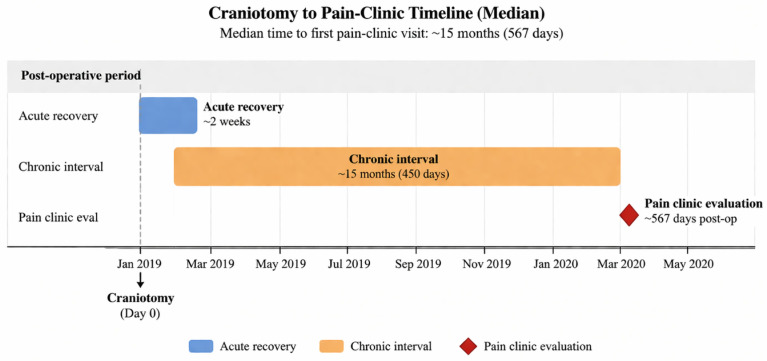
Representative timeline from craniotomy to pain clinic evaluation. This schematic depicts the typical postoperative course observed in the study cohort, characterized by an initial acute recovery phase of approximately 2 weeks, followed by a prolonged chronic interval before referral for specialized pain assessment. The median time from craniotomy to first pain clinic evaluation was approximately 15 months, underscoring delayed recognition and referral of persistent neuropathic cranial pain after surgery.

A representative timeline for a typical patient is shown in Figure 2.

The median lag of over a year (≈450 days) is consistent with under-recognition of these headaches; it underscores the need for earlier screening and referral.

Taken together, these findings suggest that post-craniotomy neuropathic pain is not a uniform condition but rather a spectrum of partially overlapping regional and scar-related syndromes. The predominance of occipital neuralgia-like pain, together with the presence of a smaller but distinct supraorbital/supratrochlear subgroup, supports the concept that the anatomical location of the surgical approach may shape the downstream neuropathic phenotype.

## 4. Discussion

In this large retrospective series, we found that neuropathic cranial pain is a distinct and not uncommon outcome of craniotomy. Approximately 2% of all craniotomy patients developed a chronic headache phenotype meeting our “probable neuropathic” criteria. This finding enriches earlier reports: while Chowdhury et al. [3] noted that 30–90% of patients report any postoperative head pain [4], our data focus on a severe, persistent subset requiring pain-specialist care. Vacas et al. [16] similarly reported that up to 30% of patients have ongoing headache, in line with our finding that 4.8% were referred for chronic pain treatment (many for non-headache reasons).

Importantly, our findings should be interpreted in the context of the institutional care pathway. Most postoperative headaches after craniotomy are managed in neurosurgical clinics by the operating surgeon and, in many cases, improve or resolve over time, as suggested by the broader post-craniotomy headache literature. By contrast, referral to a pain clinic generally occurs when pain becomes persistent, refractory, neuropathic, or neuralgiform in character. Our cohort therefore likely underestimates the overall frequency of post-craniotomy neuropathic pain, while simultaneously enriching for the very subgroup that is most clinically important to recognize. These are the patients whose symptoms are least likely to resolve spontaneously, most likely to remain under-recognized, and most relevant for preoperative counseling, prospective study, and the development of preventive strategies.

Our study corroborates and extends smaller case-based findings.

We observed clinical patterns consistent with scar-mediated neuropathic pain contributing to recalcitrant headache; surgical scalp scars can harbor neuromas, causing neuropathic pain. Allodynia at the incision site was a common feature in scar-phenotype patients. The *occipital neuralgia* subgroup mirrors Ducic’s acoustic neuroma series: chronic suboccipital lancinating pain was often associated with documented occipital nerve injury and responded to nerve blocks [14]. The overlap in 20 cases suggests that in some patients, both mechanisms coexist, for example, when a retrosigmoid or suboccipital incision affects both the regional sensory nerve territory and the scar itself as a local neuropathic generator. This “overlap syndrome” has not been explicitly described before but is biologically plausible and clinically important.

Relationship to prior literature and existing classifications

Most prior studies of post-craniotomy headache have emphasized overall headache burden, perioperative predictors, or analgesic strategies, rather than neuropathic phenotypic subclassification [21,22,23,24,25]. Our study, therefore, complements, rather than contradicts, the broader PCH literature. The approximately 2% prevalence reported here should not be interpreted as the overall prevalence of chronic headache after craniotomy; rather, it reflects the subset of patients within a referral-enriched tertiary pain pathway who met structured criteria for probable neuropathic cranial pain. In that sense, our findings identify the more refractory and clinically actionable neuropathic end of the post-craniotomy pain spectrum.

This phenotype-based approach also helps position our findings relative to existing headache classifications. Some patients in our cohort resembled exhibited features consistent with established conditions such as occipital neuralgia, whereas others demonstrated mixed postoperative syndromes with overlapping scar hypersensitivity, regional neuralgia-like pain, and partial response to targeted nerve blockade. These presentations are clinically recognizable but not always fully captured by existing formal diagnostic categories, supporting the need for pragmatic phenotype-oriented frameworks in postoperative neurosurgical populations.

### 4.1. Management Patterns

We observed that most patients received occipital nerve blocks, often with a steroid, reflecting awareness of peripheral nerve entrapment. This aligns with practice in occipital neuralgia management. Some proceeded to pulsed radiofrequency ablation of the GON/LON if blocks provided only temporary relief. Use of neuropathic pain medications was routine; our findings support guidelines advocating gabapentinoids and SNRIs as first-line treatments for neuropathic pain [15,26,27,28]. Interventional treatments (nerve decompression, neuroma excision) were rarely documented, likely due to their invasive nature and lack of neurosurgical precedent for PCH.

Importantly, the neuropathic spectrum in our cohort was not limited to posterior scalp pain. A distinct supraorbital/supratrochlear neuralgia-like subgroup emerged after frontal and frontotemporal approaches, suggesting that anterior scalp sensory branches are also vulnerable to postoperative entrapment or injury. This observation is clinically relevant because such patients may be misclassified as having non-specific frontal headache, whereas the presence of focal brow tenderness, dysesthesia, and response to supraorbital/supratrochlear block supports a peripheral neuropathic mechanism.

Mechanistically, the observed phenotypes are most consistent with a peripheral neuropathic model in at least a substantial subset of patients, including traction, transection, entrapment, or post-healing irritation of sensory scalp nerves within or adjacent to the operative field. Scar-related allodynia and focal tenderness support a local neuroma or scar-interface process, whereas response to greater occipital or supraorbital/supratrochlear blockade supports involvement of named peripheral sensory branches. At the same time, the delayed presentation and overlap across territories in some patients raise the possibility that secondary central sensitization may also contribute, particularly in longstanding, untreated cases. The relative contribution of peripheral nerve injury versus central amplification remains an important question for future prospective studies.

### 4.2. Limitations

This study has several important limitations. First, it was retrospective, single-center, and referral-based, and referral bias is therefore inherent to interpretation. Most postoperative headaches after craniotomy at our institution are managed by the treating neurosurgical team and never reach the pain clinic, particularly when symptoms are transient or improve with routine follow-up. Accordingly, our cohort likely underestimates the overall burden of post-craniotomy pain while overrepresenting the more persistent, refractory, and neuropathic/neuralgiform cases.

Second, phenotypic classification was based on chart documentation rather than prospectively applied standardized neuropathic pain instruments, quantitative sensory testing, or imaging correlates. We therefore deliberately used the term “probable neuropathic cranial pain”, and some degree of misclassification remains possible. Third, because the study was descriptive and not designed as a comparative risk-factor analysis, we cannot draw causal inferences regarding predictors such as surgical approach, pathology, sex, or age. Fourth, treatment data were available primarily as practice-pattern data, and the retrospective design did not allow robust comparative analysis of treatment efficacy, duration of benefit, or longitudinal outcome trajectories. Fifth, although we attempted to exclude pre-existing primary headache disorders when they clearly accounted for symptoms, residual confounding by migraine or other headache phenotypes cannot be fully excluded. Finally, the cohort included adults only and lacked external validation, limiting generalizability across institutions, surgeons, and pediatric populations.

### 4.3. Implications

Despite these limitations, the data underscore that neurosurgeons and pain specialists should anticipate neuropathic headaches after craniotomy. Screening for neuropathic signs (e.g., scar allodynia, trigger-point tenderness) during follow-up may allow earlier intervention. Clinically, our findings support the use of targeted treatments (nerve blocks, neuropathic medications) for the identified phenotypes. We propose our tiered inclusion/exclusion algorithm as a preliminary framework for future studies and potentially for diagnostic criteria development. For example, an “included case” would require a clear temporal relationship to craniotomy and neuropathic qualities; an excluded case would include isolated migraine recurrence. Prospective validation (with quantitative sensory testing or imaging) is needed.

Future studies should prospectively validate these phenotypes using standardized diagnostic instruments, predefined examination templates, and, where feasible, quantitative sensory testing, ultrasound or imaging correlates, and structured response assessment after targeted nerve blockade. Multicenter replication will be essential to determine generalizability across institutions and surgical approaches. In parallel, comparative analyses should examine whether specific operative corridors, pathologies, demographic factors, or perioperative variables are associated with distinct neuropathic phenotypes. Ultimately, a validated phenotype-based framework could support earlier diagnosis, more individualized treatment selection, and improved preoperative counseling for patients undergoing craniotomy.

## 5. Conclusions

In this large, single-center retrospective cohort, a clinically meaningful subset of patients developed probable neuropathic cranial pain after craniotomy, most commonly manifesting as occipital neuralgia-like pain, scar-site neuropathic pain, or, less frequently, a distinct supraorbital/supratrochlear neuralgia-like phenotype after anterior approaches. These findings support the view that persistent post-craniotomy pain is not a uniform condition but rather a spectrum of partially overlapping neuropathic syndromes that may be under-recognized in routine neurosurgical follow-up.

Although the referral-based design likely underestimates the overall prevalence of postoperative cranial pain while enriching for more refractory cases, this is precisely the subgroup of greatest clinical importance: patients whose pain persists, remains diagnostically ambiguous, and may benefit from phenotype-directed treatment. Greater awareness of scar hypersensitivity, focal nerve-territory pain, sensory disturbance, and responses to targeted nerve blockade may improve recognition of these syndromes in practice. Prospective multicenter studies are now needed to validate these phenotypes, refine diagnostic criteria, identify risk factors, and determine whether earlier targeted intervention can reduce long-term morbidity after craniotomy.

## Figures and Tables

**Table 1:** Final chart-based phenotypic classification of the neuropathic cranial pain cohort. Regional neuralgia-like phenotypes (occipital and supraorbital/supratrochlear) were analyzed alongside scar-site neuropathic pain as a partially overlapping mechanistic phenotype rather than as strictly mutually exclusive categories.

Phenotype	No. of Patients (%) Within Neuropathic Cohort (*n* = 111)	Typical Pain Territory	Typical Operative Corridor	Defining Chart-Based Features	Targeted Intervention(s)	Relationship to Scar-Site Pain
Occipital neuralgia-like	67 (60.4%)	Occipital, suboccipital, upper cervical scalp, with possible radiation to the vertex or frontal region	Retrosigmoid, suboccipital, posterior fossa, and selected non-posterior approaches with posterior scalp tract irritation	Lancinating, stabbing, or electric posterior scalp pain; focal occipital tenderness or Tinel-like hypersensitivity; C2-distribution symptoms; temporary improvement after greater and/or lesser occipital nerve block	Greater/lesser occipital nerve block; repeat nerve blocks; pulsed radiofrequency in selected refractory cases; neuropathic medications	May coexist with scar hypersensitivity or incisional pain
Supraorbital/supratrochlear neuralgia-like	8 (7.2%)	Frontal scalp, brow, supraorbital ridge, medial forehead, peri-orbital region	Frontal, frontotemporal, pterional, and anterior skull-base craniotomies	Focal frontal-orbital neuropathic pain; tenderness or Tinel-like hypersensitivity over the supraorbital/supratrochlear course; sensory disturbance in the brow/forehead; temporary improvement after supraorbital–supratrochlear blockade	Supraorbital/supratrochlear nerve block; repeat peripheral nerve blocks; neuropathic medications	Frequently overlaps with adjacent scar-site pain
Scar-site neuropathic pain	44 (39.6%)	Incision-centered scalp pain at or adjacent to the craniotomy scar	Any cranial approach	Burning, stabbing, or electric pain centered on the incision; scar allodynia; focal trigger point or painful nodularity suggestive of neuroma; localized pain provoked by touch	Local scar infiltration; trigger-point/scar injections; neuropathic medications; selected interventional procedures	Overlapping mechanistic phenotype rather than a mutually exclusive regional category
Mixed regional phenotype with scar overlap	20 (18.0%)	Combined regional neuralgia-like pain with superimposed scar hypersensitivity	Most commonly posterior or frontotemporal approaches	Coexistence of nerve-territory pain and incision-centered allodynia/hyperesthesia	Combination of regional nerve block plus scar-directed therapy; neuropathic medications	Represents phenotype overlap

## Data Availability

The data presented in this study are not publicly available due to patient privacy and institutional ethical restrictions. De-identified data may be made available from the corresponding author on reasonable request, subject to approval by the Sheba Medical Center Helsinki/Ethics Committee.
